# New Advances in Cardiorenal Syndrome—Ready for Prime Time?

**DOI:** 10.3390/jcm11123460

**Published:** 2022-06-16

**Authors:** Rainer U. Pliquett

**Affiliations:** 1 Department of Nephrology and Diabetology, Carl-Thiem Hospital Cottbus, 03048 Cottbus, Germany; rpliquett@endothel.de; 2 2nd Department of Internal Medicine, University Hospital Halle, Martin-Luther University Halle-Wittenbeg, 06108 Halle (Saale), Germany

Cardiorenal Syndrome has become one pressing issue as far as hospitalizations are concerned [1]. Even hospitalizations for chronic heart failure alone incur a subsequent increased risk for end-stage renal disease later on [2]. When using the term cardiorenal syndrome, the coincident diagnoses of chronic or acute heart failure in the presence of chronic kidney disease and/or acute kidney injury are considered as one syndrome because one organ dysfunction may affect the other one in a vicious cycle. In 2008, a classification of cardiorenal syndrome was introduced [3], which was approved by the American Heart Association in 2019 [4]. Clearly, this classification has improved the standards of cardiorenal syndrome. However, the validation studies of this classification are lacking and the clinical applicability is questioned [5]. In addition, new developments in the area of cardiorenal syndrome, such as the role of infection [6], need to be considered. Aside from hypervolemia, bradycardia; arterial hypotension; and therapy-induced hypovolemia may be regarded as a trigger for decompensated cardiorenal syndrome mediated by less cardiac output and, consecutively, by sympathoactivation leading to a centralized arterial perfusion. Hyper- or hypovolemic decompensation of cardiorenal syndrome needs to be differentiated. In the present issue of “New Advances in Cardiorenal Syndrome”, an updated classification is proposed to remedy these shortcomings [7]. This classification incorporates clinical facts potentially supporting therapeutic decisions. Clearly, this updated classification still needs to be confirmed by clinical studies, thus standing the test of time.

Aside from classification issues, both final common pathways, such as acute renin-angiotensin-aldosterone system activation, exert renal and myocardial fibrosis. An overview on the causes of “uremic cardiomyopathy” encompassing neurohumoral changes, oxidative stress and systemic inflammation is provided as an upcoming article. Speaking of systemic inflammation, contributions in this Special Issue will cover the sources of systemic inflammation in cardiorenal syndrome representing potential therapeutic targets. Last, but not least, the current perspectives provided by cardiologists and by nephrologists will cover cardiorenal syndrome as state-of-the-art articles. All contributions will discuss the challenges of diagnostics and current therapies in cardiorenal syndrome.

Many issues still remain to be resolved. Can we extrapolate the results from heart-failure studies where severe chronic kidney disease with an estimated glomerular filtration rate of less than 20 mL/min was an exclusion criteria? Do we need dedicated clinical randomized trials for cardiorenal syndrome? Do we need medical specialists for cardiorenal syndrome as well? If not, are patients with cardiorenal syndrome treated by their nephrologists or cardiologists adequately? To answer these questions, we need to take stock of the possible diagnostic and therapeutic gaps depending on what clinical path is taken. Specifically, treatment options vary depending on the perspective, e.g., the use of ventricular assist devices or the initiation of peritoneal dialysis in end-stage heart-failure patients. When hypervolemia is addressed by intermittent peritoneal dialysis, potential heart-transplant recipients may survive the waiting time by transplantation [8]. Outcome studies need to be performed comparing those different treatment options.

In the past, the majority of the published cardiologic studies on coronary artery disease required a stable kidney function and excluded chronic kidney disease, including end-stage renal disease [9]. Likewise, the majority of the clinical studies on chronic kidney disease, including end-stage renal disease, are underpowered to allow for conclusions [10]. Nevertheless, both medical therapy and specific cardiac or nephrologic interventions progressed steadily. In chronic heart failure studies, the first approved angiotensin-converting enzyme inhibitor captopril [11] was not inferior to losartan, an angiotensin receptor blocker [12,13]. Bradykinergic effects mediated by angiotensin-converting enzyme inhibition may translate into a beneficial cardiorenal outcome [14,15]. Dedicated clinical randomized trials on cardiorenal syndrome are scarce. However, in chronic, nonvalvular cardiorenal syndrome, evidence accumulated considerably. Sacubitril/valsartan, a combination of neprilysin inhibitor/angiotensin receptor blocker, was shown to be superior to the angiotensin-converting enzyme inhibitor enalapril in heart failure with reduced ejection fraction [16], associated with a lesser need for loop diuretics [17]. In addition, dapagliflozin, a sodium-glucose-cotransporter-2 inhibitor, was approved for both heart failure with reduced ejection fraction [18] and chronic kidney disease [19]. Likewise, empagliflozin was approved for chronic heart failure, regardless of the left-ventricular ejection fraction [20,21]. All in all, both the existing and upcoming results will shape the therapy of cardiorenal syndrome. The Special Issue on cardiorenal syndrome published by the Journal of Clinical Medicine aims to highlight the pathophysiological underpinnings of and current treatments for cardiorenal syndrome.
